# Preoperative anemia increases mortality and postoperative morbidity after cardiac surgery

**DOI:** 10.1186/1749-8090-9-137

**Published:** 2014-08-05

**Authors:** Antonio Miceli, Francesco Romeo, Mattia Glauber, Paolo M de Siena, Massimo Caputo, Gianni D Angelini

**Affiliations:** 1 Bristol Heart Institute, University of Bristol, Bristol BS2 8HW, UK; 2 Humanitas Clinical and Research Center, Rozzano, Milano, Italy; 3 Università Tor Vergata, Roma, Italy; 4 RUSH University Medical Centre, Chicago, USA

**Keywords:** Anemia, Cardiac surgery, Outcome

## Abstract

**Background:**

Anemia is an established adverse risk factor in cardiovascular disease. However, the effect of preoperative anemia is not well defined in heart surgery. This study evaluates the effect of preoperative anemia on early clinical outcomes in patients undergoing cardiac surgery.

**Methods:**

A retrospective, observational, cohort study of prospectively collected data was undertaken on 7,738 consecutive patients undergoing heart surgery between April 2003 and February 2009. Of these, 1,856 patients with preoperative anemia were compared to 5,882 patients without anemia (control group). According to the World Health Organization, anemia was defined as hemoglobin level < 13 g/dl for men and <12 g/dl for women. Selection bias not controlled by multivariable methods was assessed with propensity-adjustment method.

**Results:**

Overall mortality was 2.1%. Preoperative anemia was associated with tripling in the risk of death (4.6% vs 1.5%, p < 0.0001) and postoperative renal dysfunction (18.5% vs 6.5%, p < 0.0001). There was also a significant difference between the anemic and non-anemic group in the risk of postoperative stroke (1.9% vs 1.1%, p = 0.008), atrial fibrillation (36.7% vs 33%, p = 0.003) and length of hospital stay > 7 days (54% vs 36.7%, p < 0.0001). In propensity-adjusted, multivariable logistic regression, preoperative anemia was an independent predictor of mortality (odds ratio [OR] 1.44, 95% confidence interval [CI] 1.02 to 2.03), postoperative renal dysfunction (OR 1.73, 95% CI 1.43 to 2.1) and length of hospital stay > 7 days (OR 1.3, 95% CI 1.15 to 1.47).

**Conclusion:**

In patients undergoing heart surgery, preoperative anemia is associated with an increased risk of mortality and postoperative morbidity.

## Background

Anemia is an established adverse risk factor for cardiovascular disease [1]. Several studies have shown that low levels of hemoglobin are associated with increased mortality and morbidity in elderly population, in patients with congestive heart failure and coronary artery disease, especially in the setting of acute coronary syndrome [2-6]. However, the effect of preoperative anemia in cardiac surgery remains controversial. Previous investigations found preoperative anemia to be an independent risk factor of in-hospital mortality and morbidity after coronary artery bypass grafting (CABG) or valve surgery [7-12]. However, others failed to demonstrate significant differences in adverse outcomes between patients with low and normal hemoglobin levels [13,14]. Furthermore, Kulier et al. demonstrated that the presence of anemia in patients undergoing CABG was only an independent predictor of non cardiac events, whereas adverse cardiac outcomes, such as death from cardiac causes, myocardial infarction and heart failure were more related to concomitant patients’ co- morbidities rather than anemia per se [15]. Finally, the most commonly used preoperative risk stratification models in cardiac surgery, such as EuroSCORE, STS risk and ACEF, do not consider preoperative anemia a potential predictor of adverse outcome and only 2 out of 19 risk stratification models recognize anemia as an independent predictor of mortality [11,16,17]. Therefore, the aim of the present study was to evaluate the effect of preoperative anemia on early clinical outcomes in patients undergoing cardiac surgery.

## Methods

### Patients and data collection

**Figure 1:**
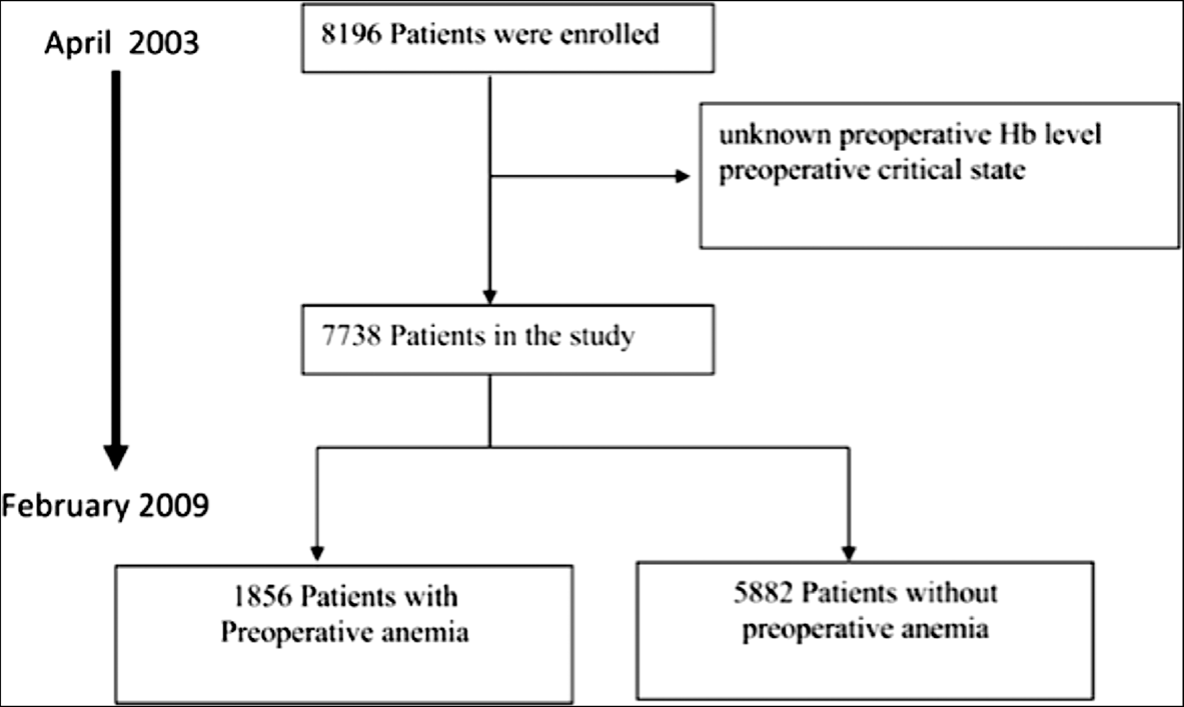
Study profile.

This was a retrospective, observational, cohort study of prospectively collected data from consecutive adult patients who underwent cardiac surgery at the Bristol Heart Institute between April 2003 and February 2009. The study was approved by the clinical audit committee of the University Hospital Bristol NHS Foundation Trust and individual consent was waived. The data collection form is entered in a database patient analysis and tracking system (Dendrite System, London, UK), and includes five section that are filled in consecutively by anaesthetists, cardiac surgeons, intensive care unit, and high dependency unit and ward nurses. The base sample contained detailed clinical information on approximately 8,196 patients. Exclusion criteria were patients who had unknown preoperative hemoglobin levels and those with pre-operative critical state, defined as any one or more of the following: ventricular tachycardia or fibrillation or aborted sudden death, preoperative cardiac massage, preoperative ventilation before arrival in the anesthetic room, preoperative inotropic support, intra-aortic balloon counterpulsation or preoperative acute renal failure (anuria or oliguria < 10 ml/hour) or emergency operations. The final sample size was 7,738 patients, of which 1,856 (24%) had preoperative anemia (Figure 1).

### End points and definitions

The primary end point was mortality, defined as any death occurring within 30 days of operation. Secondary end points were as follows: post-operative renal dysfunction (PRD), atrial fibrillation (AF), myocardial infarction (MI), stroke, and length of hospital stay.

According to the World Health Organization, anemia was defined as hemoglobin level < 13 g/dl for men and <12 g/dl for women. Priority of surgery was defined as follow: urgent (medical factors requiring the patient to stay in hospital waiting for an operation), or elective (the clinical status of the patient allows discharge from hospital with readmission for surgery at a later date). A diagnosis of post-operative MI was based on the presence of new Q waves >0.04 ms and/or a reduction in R waves >25% in at least 2 contiguous leads on electrocardiogram. The PRD was defined as a serum creatinine level >200 mol/l after surgery. New onset of postoperative AF was defined as any duration at any time in the post- operative period on the basis of a rhythm strip or 12-lead ECG. A diagnosis of stroke was made if there was evidence of new neurological deficit with morphological substrate confirmed by computed tomography or nuclear magnetic resonance imaging.

### Anaesthetic, surgical technique and postoperative management

Anaesthetic and surgical techniques were standardized for all patients and were reported previously [18]. In brief, all operations requiring cardiopulmonary bypass were conducted using a membrane oxygenator, primed with 1000 ml of Hartmanns crystalloid, 500 ml Gelofusine, 0.5 g/kg mannitol, 7 ml of 10% calcium gluconate, and 6000 IU heparin. α-Stat pH management was used, and the systemic temperature was kept between 34°C and 36°C. Myocardial protection was achieved with intermittent hyperkalaemic warm blood cardioplegia for CABG and hyperkalaemic cold blood cardioplegia for all other operations. For off-pump CABG, the Bristol technique was used to expose the coronaries and provide stabilization to undertake anastomosis [19]. At the end of surgery, patients were transferred to the intensive care unit and managed according to the unit protocol.

### Statistical analysis

Continuous data were expressed as mean ± standard deviation, and categorical data as percentages. The Kolmogorov–Smirnov test was used to check for normality of data in the two groups before further analysis. Differences between anemia and control group were compared using *χ*^2^ test for categorical variables, and the Student *t* test or the Wilcoxon rank sum test, as appropriate, for continuous variables. To reduce the effect of selection bias and potential confounding in this observational study, a propensity score was undertaken. The propensity for anemia was determined without regard of outcomes by the use of a nonparsimonious multiple logistic-regression analysis. All the variables listed in Table 1 were included in the analysis. Logistic regression was performed to assess the effect associated with anemia on each end point after adjusting for potentially confounding variables listed in Table 1 and the propensity score. Results are reported as percentages and odds ratios (OR) with 95% confidence interval (CI). All reported *P* values are two-sided, and *P* values of less than .05 were considered to indicate statistical significance. All statistical analysis was performed with SPSS 15.0 (SPSS Inc, Chicago, Ill).

**Table 1:** Patients baseline characteristics

	**Preoperative anemia (n = 1,856)**	**Control group (n = 5,882)**	**P**
Age (years ± SD)	69 ± 10.6	64.5 ± 11.6	<0.0001
Hemoglobin level (g/dl ± SD)	11.5 ± 1.1	14.3 ± 1.1	<0.0001
Female, n (%)	517 (27.9)	1419 (24.1)	<0.001
NYHA III-IV class, n (%)	809 (43.6)	1826 (31)	<0.0001
Diabetes, n (%)	424 (22.8)	703 (12)	<0.0001
Diabetes, n (%)	424 (22.8)	703 (12)	<0.0001
Hypertension, n (%)	1306 (70.4)	3910 (66.5)	0.002
Chronic Pulmonary disease, n (%)	293 (15.8)	661 (11.2)	<0.0001
Renal failure, n (%)	85 (4.6)	42 (0.7)	<0.0001
Extracardiac arteriophaty, n (%)	222 (12)	429 (7.3)	<0.0001
Previous cardiac operations, n (%)	128 (6.9)	330 (5.6)	0.046
Preoperative EF (%), n (%)			<0.0001
Good	1296 (69.8)	4579 (77.8)	
Fair	439 (23.7)	1114 (18.9)	
Poor	121 (6.5)	189 (3.2)	
Recent MI, n (%)	479 (25.8)	987 (16.8)	<0.0001
Emergency, n (%)	78 (4.2)	90 (1.5)	<0.0001
Cardiac procedures			<0.0001
CABG, n (%)	1075 (57.9)	3775 (64.2)	
Single valve surgery (n, %)	319 (17.2)	935 (15.9)	
Combined procedures (n, %)	372 (20)	655 (11.1)	
Aortic surgery, n (%)	90 (4.8)	217 (3.7)	

NYHA: New York Heart Association, EF: Ejection fraction; MI: myocardial infarction; CABG: coronary artery bypass grafting.

## Results

**Figure 2:**
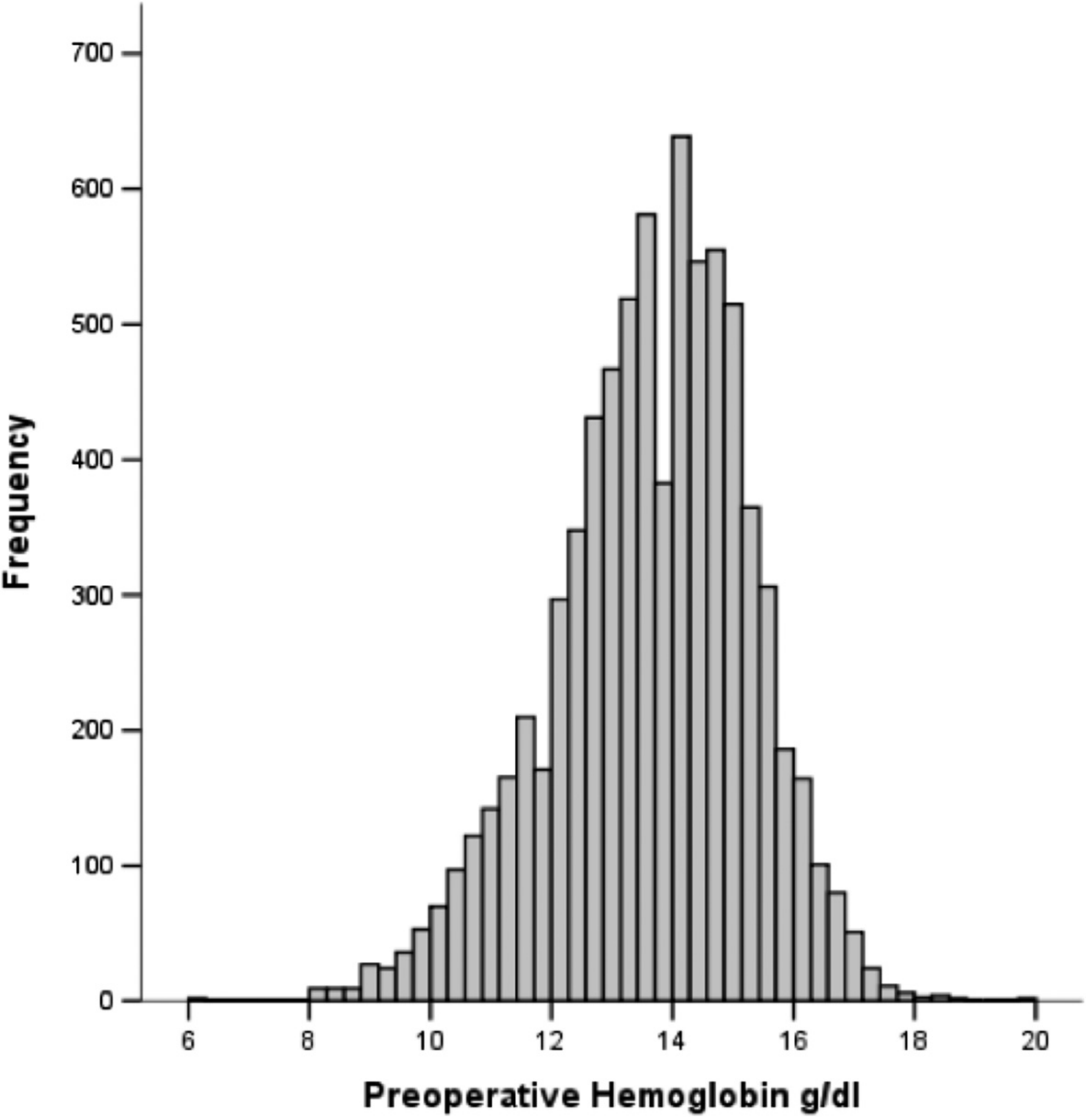
Distribution of preoperative hemoglobin levels among patients in the study.

**Figure 3:**
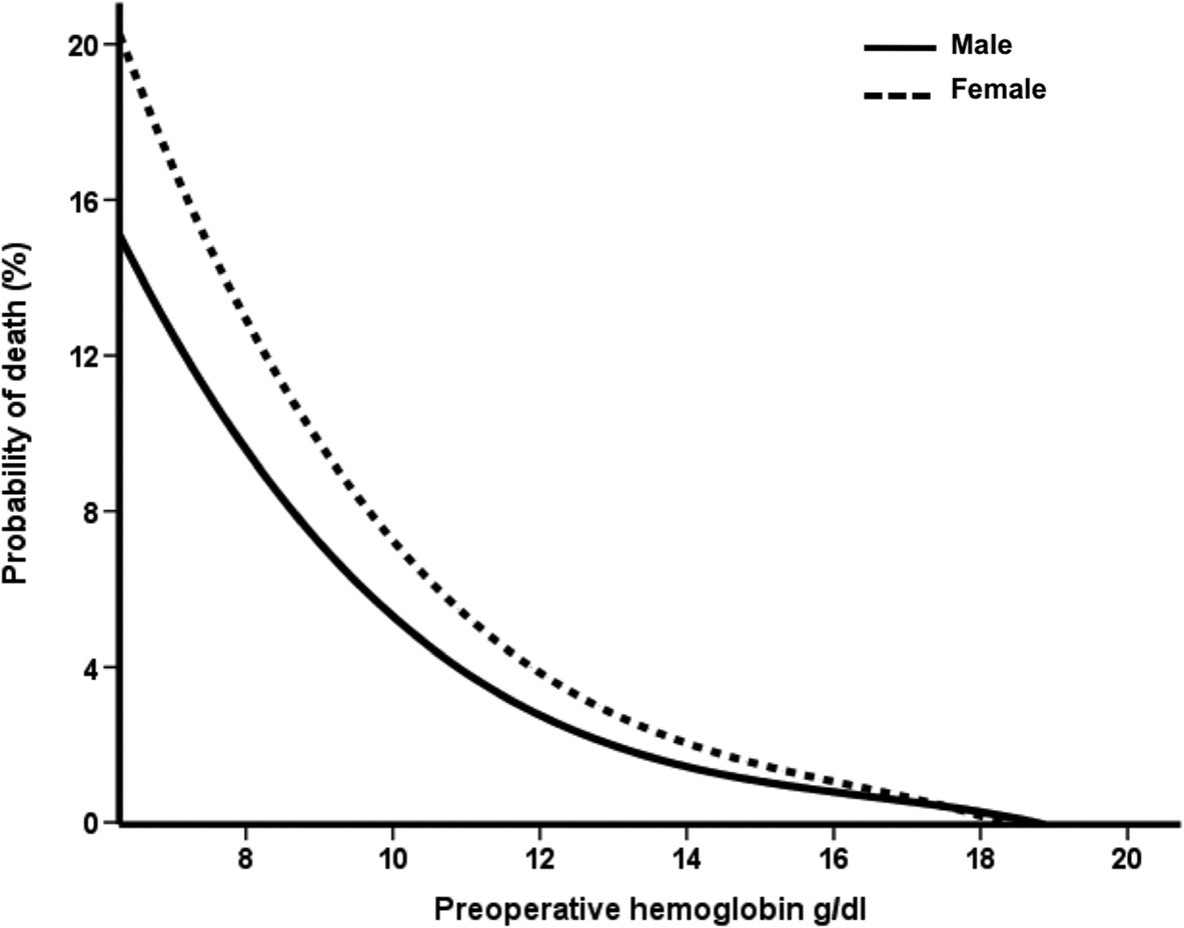
Probability of death according to preoperative hemoglobin levels.

In the study population of 7,738 patients undergoing cardiac surgery, a total of 1,856 (24%) patients had preoperative anemia (Figure 2), of which 517 (27.9%) were female. The mean (±standard deviation, SD) hemoglobin concentration was 11.5 (SD, 1.1) g/dl versus 14.3 (SD, 1.1) g/dl, in the anemic and control patients group, respectively. Baseline characteristics of the study population are shown in Table 1. Patients in the anemia group were older and more often female; they were more likely to have a greater prevalence of hypertension, chronic obstructive pulmonary disease, chronic kidney disease, extracardiac arteriophaty, and previous cardiac operations. Patients with anemia were also more likely to have a lower ejection fraction and greater prevalence of New York Heart Association functional class. Finally, the prevalence of patients undergoing other than isolated CABG and aortic surgery were higher in the anemic patients than in the control group. The overall rate of mortality was 2.1%. Results from univariate and multivariable logistic analysis are reported in Tables 2 and 3. Preoperative anemia was associated with a three-fold increased in the risk of death (4.6% vs 1.5%, p < 0.0001) and postoperative renal dysfunction (18.5% vs 6.5%, p < 0.0001). There was also a significant difference between the anemic and non-anemic group in the risk of postoperative stroke (1.9% vs 1.1%, p = 0.008), atrial fibrillation (36.7% vs 33%, p = 0.003) and length of hospital stay > 7 days (54% vs 36.7%, p < 0.0001. There was no significant difference in postoperative myocardial infarction (1.9% vs 2%, p = 0.93) between the two groups. After adjusting for confounding variables listed in Table 1, as well as propensity score, preoperative anemia was an independent predictor of mortality, postoperative renal dysfunction and length of hospital stay > 7 days. Finally, when modeled as univariate continuous variable, a curvilinear relationship was seen between the estimated probability of death and preoperative hemoglobin either in the female or in the male population (Figure 3).

**Table 2:** Clinical outcomes

	**Anemia group N = 1856**	**Control group N = 5882**	***p***
Mortality, % (n)	76 (4.6)	88 (1.5)	<0.0001
Atrial fibrillation, % (n)	682 (36.7)	1942 (33)	0.003
Renal dysfunction, % (n)	344 (18.5)	384 (6.5%)	<0.0001
Stroke, % (n)	36 (1.9)	65 (1.1)	0.008
Myocardial infarction, % (n)	36 (1.9)	118 (2)	0.93
Length of hospital stay >7 days, % (n)	1002 (54)	2159 (36.7)	<0.0001

**Table 3:** Observed and adjusted ORs of postoperative adverse outcomes after cardiac surgery, according to the presence or absence of anemia

**Outcome**	**Crude OR (95% CI)**	Adjusted* OR (95%)
Mortality	2.81 (2.06-3.84)	1.44 (1.02-2.03)
Atrial fibrillation	1.18 (1.06-1.31)	0.96 (0.85-1.08)
Renal dysfunction	3.26 (2.8-3.8)	1.73 (1.43-2.1)
Stroke	1.77 (1.17-2.67)	0.95 (0.61-1.47)
Myocardial infarction	0.967 (0.66-1.4)	0.79 (0.53- 1.19)
Length of hospital stay >7 days	2.03 (1.82–2.25)	1.3 (1.15–1.47)

*Adjusted for baseline characteristics and propensity score.

## Discussion

Our study demonstrates that preoperative anemia defined by the World Health Organization as hemoglobin level <13 g/dl for men and <12 gr/dl for women, is a risk factor for mortality and morbidity in patients undergoing cardiac surgery. Low levels of hemoglobin were associated with tripling in the risk of death and postoperative renal dysfunction. Furthermore, patients with preoperative anemia had a 30% increased risk of prolonged hospital stay > 7 days. We did not observe any significant relationship between anemia and the risk of myocardial infarction. Finally, as shown in Figure 3, the estimated probability of death increased with decreasing levels of preoperative hemoglobin.

Anemia is an established risk factor for cardiovascular disease in the general population [1,2]. The authors of the ARIC (Atherosclerosis Risk in Communities) study analyzed 14,410 low risk subjects in the general population between the ages of 45 and 64 years and suggested that anemia was associated with increased risk in the total rate of myocardial infarction, coronary angioplasty, coronary artery bypass surgery or heart disease death [1]. Moreover, several studies have shown that low levels of hemoglobin were independent predictors of mortality and morbidity in patients with heart failure and coronary artery disease [3-6]. In a cohort of 12,065 patients with new-onset heart failure, Ezekowitz showed a 34% increased risk in mortality associated with anemia [3]. Furthermore, Sabatine et al. in a review of 39,922 patients from 16 randomized trials, demonstrated that patients with ST- elevation MI had a progressive increase in cardiovascular mortality and heart failure as the baseline hemoglobin dropped below 14 g/dl, whereas subjects with non ST-elevation MI had an increased odds of cardiovascular death, MI, and recurrent ischemia as the baseline hemoglobin fell below 11 g/dl [5].

Anemia is the most common hematological problem in the preoperative patient and according to the definition used, its incidence ranges from 22% to 30% and over 40% in octogenarians [8-14]. In our sample, one quarter of patients undergoing surgery were anemic. A number of studies have shown a significant association between anemia and adverse outcomes after surgery [20-22]. Wu et al. analyzed the effect of preoperative hematocrit levels in over 310,000 elderly veterans undergoing non-cardiac surgery and found that even mild anemia was associated with an increased risk of 30-day mortality and morbidity [20]. In addition, they showed a linear rise in death and cardiac events when the hematocrit level was less than 39% [20]. Similar results are reported in patients undergoing cardiac surgery [7-13]. In a large multicenter cohort study on 3,500 patients, Karkuti et al. found that preoperative anemia was independently associated with doubling in the composite risk of in-hospital death, stroke, or acute kidney injury; the same odds were even obtained after adjusting for confounders by propensity score matching [8]. Using the same statistical technique, Ranucci et al. demonstrated that patients with any degree of anemia had a significantly higher rate of stroke, major morbidity and higher operative mortality rate [12]. Zindrou et al. observed that individuals with a preoperative hemoglobin concentration of 10 g/dl or less had a 3-fold increase in the odds of in-hospital mortality after CABG than those with a higher hemoglobin concentration [7]. Finally, in the setting of cardiac valve surgery, Clandellas et al. found that patients with preoperative hemoglobin <12 mg/dl had a 3-fold increased risk of death and 5-fold in the odds of developing major postoperative complications such as, heart failure, myocardial infarction, reoperation for bleeding, neurological complication and acute renal failure [10]. Nevertheless, other studies failed to show a negative impact of preoperative anemia on adverse postoperative outcomes. Bell et al. and Carrascal et al. demonstrated that low preoperative hemoglobin level was not an independent risk factor for early mortality after CABG and that it was only mildly significant for morbidity even in octogenarian patients [13,14]. In addition, Kulier et al. demonstrated that the presence of low hemoglobin levels did not predict postoperative cardiac outcomes, such as myocardial infarction, congestive heart failure or death from cardiac causes, but only renal and cerebral adverse complications [15]. The different results reached by these authors might be partially explained by the threshold chosen for the definition of preoperative anemia. These studies used cutoff points for preoperative hemoglobin that ranged from 10 to 12.5 g/dl and, most of them focused only on single isolated procedures. On the contrary, the strength of our investigation was to define anemia according to the World Health Organization and evaluate prospectively collected data from a large number of consecutive patients undergoing different cardiac surgery operations.

Finally, the best known risk stratification tools do not consider low hemoglobin level a potential predictor of adverse outcome [16]. These controversies have raised the question of whether preoperative anemia is a marker of other concomitant comorbidities such as advanced age, diabetes and renal dysfunction, or a risk factor “per se”. According to Ranucci et al., anemia is often excluded from the risk model because of the low prevalence of anemic patients in the examined populations and the strong correlation (multicollineratity) between low hemoglobin levels and other comorbidities [12]. In our sample, one quarter of patients were anemic and we demonstrated that anemia was still an independent predictor of mortality and morbidity after adjusting for baseline characteristics and propensity score.

The association between preoperative anemia and adverse outcomes in patients after cardiac surgery may have several explanations. First, anemic patients are at risk for inadequate tissue oxygenation delivery during the peri-operative period, which may lead to impaired tissue oxygenation and organ dysfunction. Second, anemia is the most important predictor of hemodilution during cardiopulmonary bypass. It has been shown that low hematocrit levels are independently associated with mortality, stroke and acute renal failure [12,23-25].

Finally, anemia is the strongest predictor of blood transfusions [21,26]. It has been shown that blood cell transfusions in patients having cardiac surgery are strongly associated with increased mortality and morbidity [27]. These results might be related to the functional and structural changes that occur in stored blood cells. The increased red cell aggregability and rigidity, the accumulation of potentially toxic microparticles or proinflammatory cytokines as well as the depletion of NO in the stored blood might promote vasoconstriction, platelet aggregation and ineffective oxygen depletion, causing tissue hypoxia and organ dysfunction [27-29].

Despite of low levels of hemoglobin have been associated with worse results, preoperative anemia is a risk factor that can be modified by treatment before surgery. In this regard, the updated guidelines of the Society of Thoracic Surgeons and the Society of Cardiovascular Anesthesiologists Blood Conservation recommend the preoperative administration of erythropoietin and iron therapy with the aim of increasing hemoglobin levels (class II a, level of evidence B) [30]. Finally, it has been proposed that prophylactic blood transfusions might limit any subsequent deleterious effects by stabilizing the iron metabolism and production of oxidative reaction before surgery come into play [31]. However, more studies are required to confirm these data.

### Study limitation

This study is based on the retrospective analysis of our large institutional observational prospectively collected database, and we are unable to account for the influence of any residual unmeasured factors that could affect the adverse outcomes. Furthermore, we did not take into account specific details on the intraoperative and postoperative management of these patients and in particular information regarding the minimum Ht level during cardiopulmonary bypass, postoperative blood loss and blood transfusions.

## Conclusions

In patients undergoing cardiac surgery, preoperative anemia, defined as hemoglobin level < 13 g/dl for men and <12 g/dl for women, is an independent risk factor for mortality, postoperative renal dysfunction, stroke and prolonged hospital stay.

## Abbreviations

CABG: Coronary artery bypass grafting; PRD: Post-operative renal dysfunction; AF: Atrial fibrillation; MI: Myocardial infarction; OR: Odds ratio; CI: Confidence interval; SD: Standard deviation.

## Competing interest

The authors declare that they have no competing interests.

## Authors’ contributions

AM: has participated in the conception and design of study, acquisition of data, analysis and interpretation of data; and has been involved in drafting of the manuscript. FR: agrees to be accountable for all aspects of the work in ensuring that questions related to the accuracy or integrity of any part of the work are appropriately investigated and resolved. MG: have been involved in revising the manuscript critically for important intellectual content and in interpretation of data. PMdS: : has participated in the conception and design of study, acquisition of data, has been involved in the revision of the manuscript. MC: has participated in the conception and design of study, acquisition of data, interpretation of data; and has been involved in drafting of the manuscript. GDA: has been involved in revising the manuscript critically for important intellectual content and has given final approval of the version to be published. Every author takes responsibility for all aspects of the reliability and freedom from bias of the data presented and their discussed interpretation. All authors read and approved the final manuscript.
